# FTY720 ameliorates GvHD by blocking T lymphocyte migration to target organs and by skin fibrosis inhibition

**DOI:** 10.1186/s12967-020-02386-w

**Published:** 2020-06-06

**Authors:** Jaeyoon Ryu, Jooyeon Jhun, Min-Jung Park, Jin-ah Baek, Se-Young Kim, Keun-Hyung Cho, Jeong-Won Choi, Sung-Hwan Park, Jong Young Choi, Mi-La Cho

**Affiliations:** 1 The Rheumatism Research Center, Catholic Research Institute of Medical Science, College of Medicine, The Catholic University of Korea, Seoul, Republic of Korea; 2 Division of Rheumatology, Department of Internal Medicine, Seoul St. Mary’s Hospital, College of Medicine, The Catholic University of Korea, Seoul, South Korea; 3 Department of Medical Lifescience, College of Medicine, The Catholic University of Korea, 222, Banpo-daero, Seocho-gu, Seoul, 06591 Republic of Korea; 4 Division of Hepatology, Department of Internal Medicine, College of Medicine, Seoul St. Mary’s Hospital, The Catholic University of Korea, Seoul, 137-040 South Korea

**Keywords:** FTY720, Sphingosine-1-phosphate (S1P), Graft-versus-host disease, Skin fibrosis, Th17

## Abstract

**Background:**

Fibrosis is the formation of excess connective tissue in an organ or tissue during a reparative or reactive process. Graft-versus-host disease (GvHD) is a medical complication of allogeneic tissue transplantation with transplanted donor T cell-mediated inflammatory response; it is characterized by a severe immune response with fibrosis in the final stage of the inflammatory process. T helper 17 cells play a critical role in the pathogenesis of GvHD. Fingolimod (FTY720), an analogue of sphingosine-1-phosphate (S1P), is an effective immunosuppressive agent in experimental transplantation models.

**Methods:**

In this study, we evaluated the effects of FTY720 as a treatment for an animal GvHD model with inflammation and fibrosis. The splenocytes, lymph nodes, blood, tissues from Syngeneic mice and GvHD-induced mice treated vehicle or FTY720 were compared using flow cytometry, hematological analyses, histologic analyses.

**Results:**

FTY720 reduced clinical scores based on the following five clinical parameters: weight loss, posture, activity, fur texture, and skin integrity. FACS data showed that T lymphocyte numbers increased in mesenteric lymph nodes and decreased in splenocytes of FTY720-treated mice. Tissue analysis showed that FTY720 reduced skin, intestinal inflammation, and fibrotic markers. FTY720 dramatically decreased α-smooth muscle actin, connective tissue growth factor, and fibronectin protein levels in keloid skin fibroblasts.

**Conclusions:**

Thus, FTY720 suppressed migration of pathogenic T cells to target organs, reducing inflammation. FTY720 also inhibited fibrogenesis marker expression in vitro and in vivo. Together, these results suggest that FTY720 prevents GvHD progression via immunosuppression of TH17 and simultaneously acts an anti-fibrotic agent.

## Background

Allogeneic hematopoietic stem cell transplantation (allo-HSCT) is a widely used immunotherapy for hematologic malignancies. Although allo-HSCT is an effective treatment, its application has been limited by graft-versus-host disease (GvHD), a critical complication of allo-HSCT. In GvHD, recipient antigen-presenting cells (APCs) recognize donor T cells as antigens, thereby inducing the migration of pathogenic T lymphocytes to target organs and a systemic inflammatory condition in which cytokines that aggravate disease symptoms are secreted [1, 2]. Initiation and development of chronic GvHD follows a 3-phase model. As GvHD progresses, tissue injury propagation increases due to dysregulated activity of pathogenic T cells and an irregular repair system, inducing fibroblast activation and excessive accumulation of extracellular matrix. The resulting continuous aberrant immunity and tissue repair system can lead to scarring or fibrosis [3]. Fibrosis is characterized by excessive accumulation of organ connective tissues as a result of dysfunctional wound-healing in response to chronic inflammation. Fibrosis can affect all tissues throughout the body. When highly aggravated, fibrogenesis leads to organ failure and death [4, 5]. Therefore, a treatment strategy is needed to address all of the effects of GvHD simultaneously.

Fingolimod (FTY720) is a well-known immune modulator that is used to treat multiple sclerosis [6]. FTY720 is a structural analogue of sphingosine and targets receptors for sphingosine-1-phosphate (S1P) including S1P_1_, a potent signaling molecule. The high density of S1P receptors on the lymphocyte surface induces their migration from lymph nodes to target organs. Accordingly, S1P_1_ plays a significant role in the egress of pathogenic T lymphocytes from lymph nodes in immune diseases [7]. FTY720 traps naïve and antigen-activated CD4^+^ T cells and blocks lymphocyte migration to target organs through S1P_1_ internalization, inducing T cell accumulation in lymph nodes [8–11]. In a past study, it reveals that pretreatment of FTY720 before transplantation was sufficient to improve GvHD because of reducing host DCs in recipient spleen and FTY720 blocked expansion of donor CD4+ and CD8+ effector T cells after transplantation. [12] Furthermore, FTY720 effectively suppressed liver fibrosis through the prevention of bone marrow-derived mesenchymal stem cells (BMSCs) migration in the carbon tetrachloride (CCL4)-induced mouse model. [13] FTY720 inhibited expression of the cytokines that have been shown to play a role in liver fibrosis in the mice model of diet-induced non-alcoholic fatty liver disease while FTY720 only had a minimal effect on inflammation. [14] Early administration of FTY720 inhibited the severity and fibrosis in murine sclerodermatous chronic GvHD through decreasing soluble collagen and dermal thickness. [15] In a recent study, FTY720 was found to directly and specifically target various phenotypes of hypertrophic scarring fibroblasts (HSFs), suppressing the expression of fibrotic markers such as α-smooth muscle actin (α-SMA) and collagen, effectively suppressing HSF activation [16].

In this study, we hypothesized that FTY720 simultaneously regulates T cell migration and fibrogenesis. We tested the anti-allo-response capacity of FTY720 via the alloreactive T cell response in vitro. We then investigated whether FTY720 inhibits GvHD using a bone marrow transplantation (BMT) mouse model. We further examined whether FTY720 suppresses fibrotic factors in vitro and in vivo.

## Methods

### Alloreactive T cell responses in vitro

Responder cells, or CD4^+^ T cells, were derived from splenocytes of Balb/c (B/c) mice using MACS separator after 15 min incubated with CD4 (L3T4) MicroBeads (Miltenyi Biotec, San Diego, CA, USA) at 4 °C. Splenocytes isolated from B6 mice acted as APCs with respect to allorecognition; we therefore collected non-T cells with negative separation method after the same process as responder cells. All the cells maintained in RPMI medium containing 5% fetal bovine serum (FBS). APCs were irradiated at 3000 cGy and seeded with responder cells not irradiated (1 × 10^5^); irradiated APCs were cultured in 96-well plates for 3 days and cells were treated with vehicle (Dimethyl sulfoxide; DMSO) diluted in the media the same proportion as the highest concentration of FTY720 for the control condition, and with or without FTY720 (1 or 5 μM) for the other conditions, pulsed with 1 μCi tritiated thymidine (3[H]-TdR; NEN Life Science Products Inc., Boston, MA, USA) 16 h before the end of the experiment, and counted using a PHD cell harvester (Cambridge Technology, Inc., Cambridge, MA, USA). APCs from B/c mice and CD4^+^ T cells from B6 mice were counted following the same procedure. Data are expressed as mean counts per minute of triplicate samples ± standard deviation (SD).

### Alloreactive human peripheral blood mononuclear cell (PBMC) response in vitro

Blood samples were derived from healthy donors, and PBMCs were isolated. To obtain PBMCs, blood from each donor was mixed with phosphate-buffered saline (PBS), and the mixture was floated carefully onto a layer of 10 mL Ficoll Plaque plus (GE Healthcare Life Sciences, Marlborough, MA, USA). Samples were centrifuged for 30 min at 2000 rpm at 20 °C to divide blood contents. PBMCs collected after centrifugation were washed with PBS and maintained in RPMI medium containing 10% fetal bovine serum (FBS). To observe the alloreactive human PBMC response, two blood samples from healthy donors were examined. One sample acted as a responder and the other as a stimulator, similar to APCs. Stimulator PBMCs (1 × 10^5^) were irradiated at 5000 cGy and seeded with responder PBMCs not irradiated (1 × 10^5^) into 96-well plates for 3 days and cells were treated with vehicle (dimethyl sulfoxide; DMSO) diluted in the media the same proportion as the highest concentration of FTY720 for the control condition, and with or without FTY720 (1 or 5 μM) for the other conditions; they were then pulsed with 1 μCi 3[H]-TdR 16 h before the end of the experiment. Harvesting and measurement were conducted as for the alloreactive mouse T cell response experiment.

### Cytokine level measurement by enzyme-linked immunosorbent assay (ELISA)

Concentrations of interleukin (IL)-17, and IL-10 in culture supernatants from alloreactive human PBMC response were measured using an ELISA DuoSet (R&D Systems, Lille, France).

### Mouse bone marrow transplantation (BMT)

Nine-week-old C57BL/6 (B6) (H-2 kb) and B/c (H-2kd) mice were purchased from Orient Bio (Seongnam, Korea). Mice were maintained under specific pathogen-free conditions in an animal facility at 22 ± 1 °C, 55 ± 5% humidity, and a light/dark cycle of 12 h/12 h. The air in the facility was passed through a high-efficiency particulate air (HEPA) filter system to exclude bacteria and viruses. Animals were fed mouse chow and tap water ad libitum. The protocols used in this study were approved by the Animal Care and Use Committee of the Catholic University of Korea. To develop the GvHD model, splenocytes (5 × 10^6^) and bone marrow cells (5 × 10^6^) were isolated from B6 donor mice and transplanted into B/c recipient mice via intra-vein (i.v.) injection. Before transplantation, B/c mice were sub-lethally irradiated at 690 cGy and allowed to rest for a couple of hours. Following the induction of GvHD, FTY720 was administered orally to recipient mice daily, commencing on day 0 after BMT (0.5, 1, and 3 mg/kg/days per mouse). Control GvHD mice received vehicle (saline), administered in the same manner as the treatment group. 10 mice were used in each groups. All experimental procedures were approved by the Department of Laboratory Animals, Institutional Animal Care and Use Committee (IACUC) of the School of Medicine, Catholic University of Korea and conformed with all National Institutes of Health (USA) guidelines (Permit Number: CUMC 2018-0168-04).

### Survival and histopathology scoring

Survival after BMT was monitored daily and the extent of clinical GvHD was assessed weekly using a scoring system that summed changes in five clinical parameters: weight loss, posture, activity, fur texture, and skin integrity. Mice irradiated at 690 cGy were euthanized 28 days after BMT prior to blinded histopathology of GvHD target tissues.

### Hematological analysis

Blood was collected from the heart via cardiac puncture in BD Microtainer Tubes with K2E (Becton–Dickinson and Co., Franklin Lakes, NJ, USA). Hematological parameters were recorded using a Hemavet 950 hematology system (Drew Scientific, Cumbria, UK).

### Flow cytometry

Mouse lymphocytes from mesenteric lymph nodes and spleens were immunostained using fluorescently conjugated antibodies against CD4 (eBioscience, San Diego, CA, USA), IL-17 (eBioscience), interferon (IFN) γ (BioLegend, San Diego, CA, USA), and IL-4 (BD Biosciences, San Diego, CA, USA). Prior to intracellular staining, cells were stimulated for 4 h with phorbol myristate acetate (25 ng/mL) and ionomycin (250 ng/mL) in the presence of Golgistop (BD Biosciences). Intracellular staining was performed using a BD Cytofix/Cytoperm Plus Fixation/Permeabilization Kit and BD Golgistop Kit (BD Biosciences). The transcription factor Foxp3 was stained using a Foxp3/Transcription Factor Staining Kit (eBioscience) following the manufacturer’s protocol. Flow cytometry was performed with the aid of a cytoFLEX Flow Cytometer (Beckman Coulter, Brea, CA, USA).

### Histopathological and immunohistochemical analyses and immunofluorescence staining

Formalin-fixed skin, liver, and large and small intestinal tissue sections were paraffin-embedded and stained with hematoxylin and eosin. Epithelial loss, crypt damage, goblet cell depletion, and inflammatory cell infiltration were histologically scored [17]. Paraffin-embedded sections were subjected to immunohistochemistry analysis using a Vectastain ABC kit (Vector Laboratories, Burlingame, CA, USA). Immunohistochemistry were performed on tissue sections of all mice (n = 30) of 3 groups. Three slides were prepared for each sample per mice and each slide was taken at least 500 μm apart. Immuno-stained sections were examined by a photomicroscope (Olympus, Tokyo, Japan). The number of positive cells was counted at high-power field (magnifications: 400×) with the aid of Adobe Photoshop software and averaged 3 randomly selected fields per tissue section. Sections were incubated with primary antibodies against α-SMA (Abcam, Cambridge, UK), Collagen 1 (Abcam), and Fibronectin (FN1) (Abcam, Cambridge, UK) overnight at 4 °C, followed by addition of a biotinylated secondary antibody and a streptavidin-peroxidase mixture for 1 h. Color was developed by addition of 3,3-diaminobenzidine (Dako, Carpinteria, CA, USA). Tissue sections in paraffin molds were stained with connective tissue stain using a Picro Sirius Red Stain Kit (Abcam).

### Primary fibroblast cell culture

Keloid tissues were washed with PBS, cut into small pieces, and adhered and cultured on the bottoms of tissue culture flasks. After 3 days of culture, fibroblasts grew from the tissues. Primary keloid fibroblasts were collected and cultured in Dulbecco’s Modified Eagle Medium (Gibco, Grand Island, NY, USA) supplemented with 10% FBS (Gibco), containing 100 μg/mL streptomycin and 100 U/mL penicillin, at 37 °C in an atmosphere of 5.0% CO_2_. Primary keloid fibroblasts used in this study were retained in the 7th to 11th passages.

### Western blot analysis

Primary keloid fibroblasts were washed with PBS and harvested for total protein extraction using lysis buffer (Applied Science, Mannheim, Germany). Cell lysates were centrifuged for 15 min at 4 °C to pellet cellular debris. Proteins were separated by 10% sodium dodecyl sulfate polyacrylamide gel electrophoresis and transferred to nitrocellulose membranes (Invitrogen Life Technologies, Carlsbad, CA, USA). Membranes were incubated with primary antibodies specific to α-SMA (Abcam), connective tissue growth factor (CTGF) (Abcam), Fibronectin-1 (Abcam), and β-actin (Santa Cruz Biotechnology). Primary antibodies were detected using goat anti-mouse or anti-rabbit horseradish peroxidase-conjugated secondary antibodies. The resulting bands were revealed using enhanced chemiluminescence reagents (Amersham Biosciences, Piscataway, NJ, USA).

### Statistical analyses

We performed one-way analysis of variance (ANOVA) with Bonferroni’s post hoc test for multiple comparisons, at a significance level of p < 0.05. GraphPad Prism ver. 5.01 software (GraphPad Software Inc., San Diego, CA, USA) was used for all analyses. Data are presented as mean ± SD.

## Results

### FTY720 suppressed alloreactive T cell proliferation

**Fig. 1:**
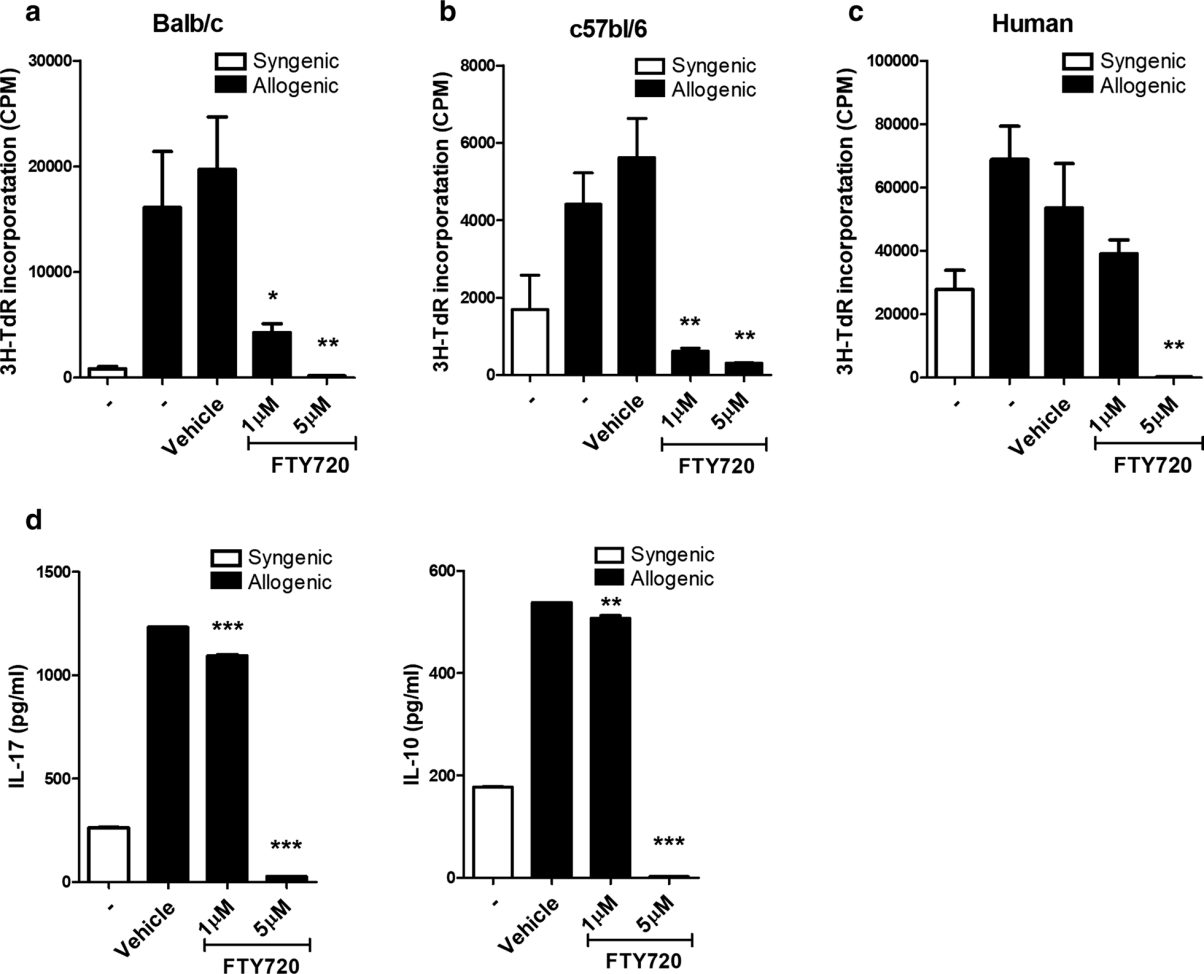
FTY720 suppressed Alloreactive T-cell responses in mice and human with reducing pro-inflammatory cytokines. **a**, **b** In the mixed lymphocyte reaction assay, a total of 10^5^ B/c(B6) splenic T cells (responders) were incubated with 10^5^ irradiated B/c(B6) (syngeneic stimulators [Syn]) or B6(B/c) (allogeneic stimulators [Allo]) splenic APCs for 4 days. **c** In the mixed lymphocyte reaction assay, human PBMC of healthy volunteer were incubated with another volunteer for 4 days. **d** IL-17 and IL-10 concentrations in culture supernatants as determined by ELISA

To determine whether FTY720 reduces allo-response, a well-known effect of FTY720, we measured T cell proliferation in a mixed lymphocytes reaction (Fig. 1a, b). To investigate the alloreactive human PBMC response, two blood samples from healthy donors were also examined (Fig. 1c) and the cultured supernatants were analyzed by ELISA (Fig. 1d). Importantly, we detected dose-dependent allo-response repression. Especially, 5 μM of FTY720 almost completely suppressed allo-response. ELISA results also showed that 5 μM of FTY720 inhibited the secretion of IL-17, a well-known pro-inflammatory cytokine, and IL-10 known as anti-inflammatory cytokine while vehicle has no suppression effects. These findings suggest an inhibitory function of FTY720 against T cells in vitro.

### FTY720 ameliorated GvHD disease severity in a mouse model

**Fig. 2:**
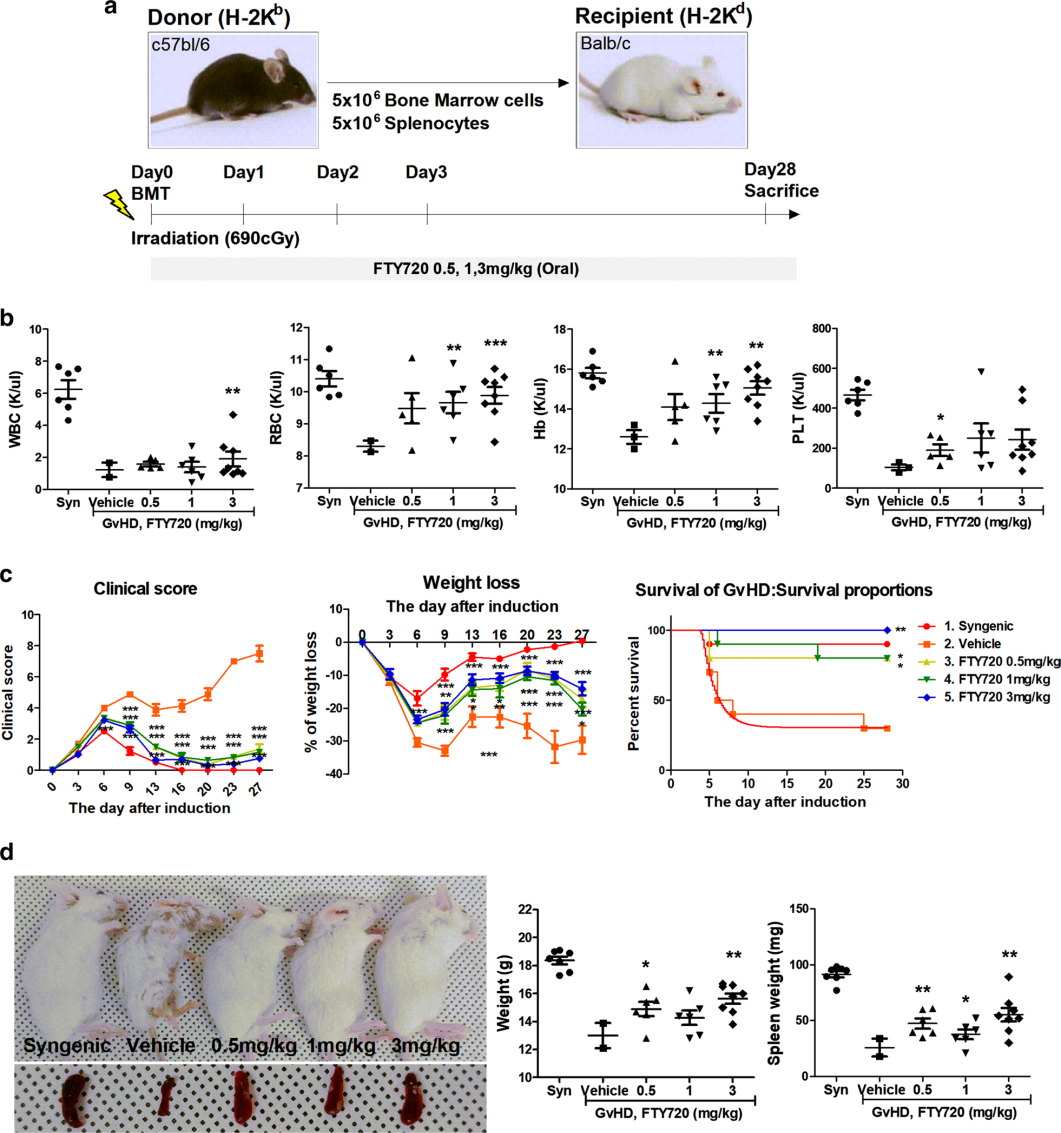
FTY720 upregulated hematocytes in Hematological analysis of peripheral blood, recovering symptoms of GvHD mice. **a** Female Balb/c recipients received 0.5, 1, 3 mg/kg FTY720 per mouse(20 g) beginning at day 0. For allo-HSCT, total BM cells and splenocytes from C57BL/6 were given by tail vein injection. Recipients were given with 690 cGy before injection. The GvHD-induced mice were sacrificed at day 28. **b** Hematological analysis of peripheral blood at day 28. **c** Clinical score after BMT, Weight loss in acute GvHD phase, and Survival after BMT. Allogeneic-transplanted animals showed significant increased GvHD score compared with control animals. Animals were regularly scored for five clinical parameters (weight loss, posture, activity, fur and skin) on a scale from 0 to 2. Clinical GvHD score was generated by summation of these five parameters. **d** Phenotype of syngeneic- and allogeneic-transplanted mice and Spleen at day 28 (the day of sacrifice)

FTY720 significantly inhibited T cell proliferation (Fig. 1). We therefore induced a GvHD mouse disease model to ascertain whether FTY720 has the same efficacy in vivo. C57BL/6 (B6; H-2kb) and B/c (H-2kd) mice were used in this model. To induce the GvHD model, splenocytes (5 × 10^6^) and bone marrow cells (5 × 10^6^) were separated from B6 donor mice and transplanted into B/c recipient mice via i.v. injection. B/c mice were sublethally irradiated at 690 cGy before transplantation. FTY720 was administered daily at 0.5, 1, and 3 mg/kg for 28 days from the day of induction (day 0) (Fig. 2a). We performed hematological analysis of the GvHD mouse model via heart puncture and analysis using an automated blood analyzer. Hematological indicators recovered in a dose-dependent manner included white blood cells, red blood cells, hemoglobin, and platelets (Fig. 2b). FTY720 administration effectively improved clinical symptoms including weight loss, posture, activity, fur texture, and skin integrity after day 13, compared with the vehicle group. Regardless of dosage, clinical scores including weight loss showed that FTY720 treatment groups had recovered significantly. The 3-mg/kg FTY720 treatment group showed zero mortality, whereas two mice died in the vehicle-treated group on day 5, and only three mice survived to the end of the experiment (day 28) (Fig. 2c). Mice in the vehicle group were smaller and weighed less than FTY720-treated mice, and exhibited fur loss, reddened skin in bald patches, hunched posture, and overall poor condition; FTY720-treated mice recovered from signs of GvHD. Spleen size and weight improved in FTY720-treated mice by the day of sacrifice (day 28) (Fig. 2d).

### FTY720 trapped T lymphocytes in mesenteric lymph nodes

**Fig. 3:**
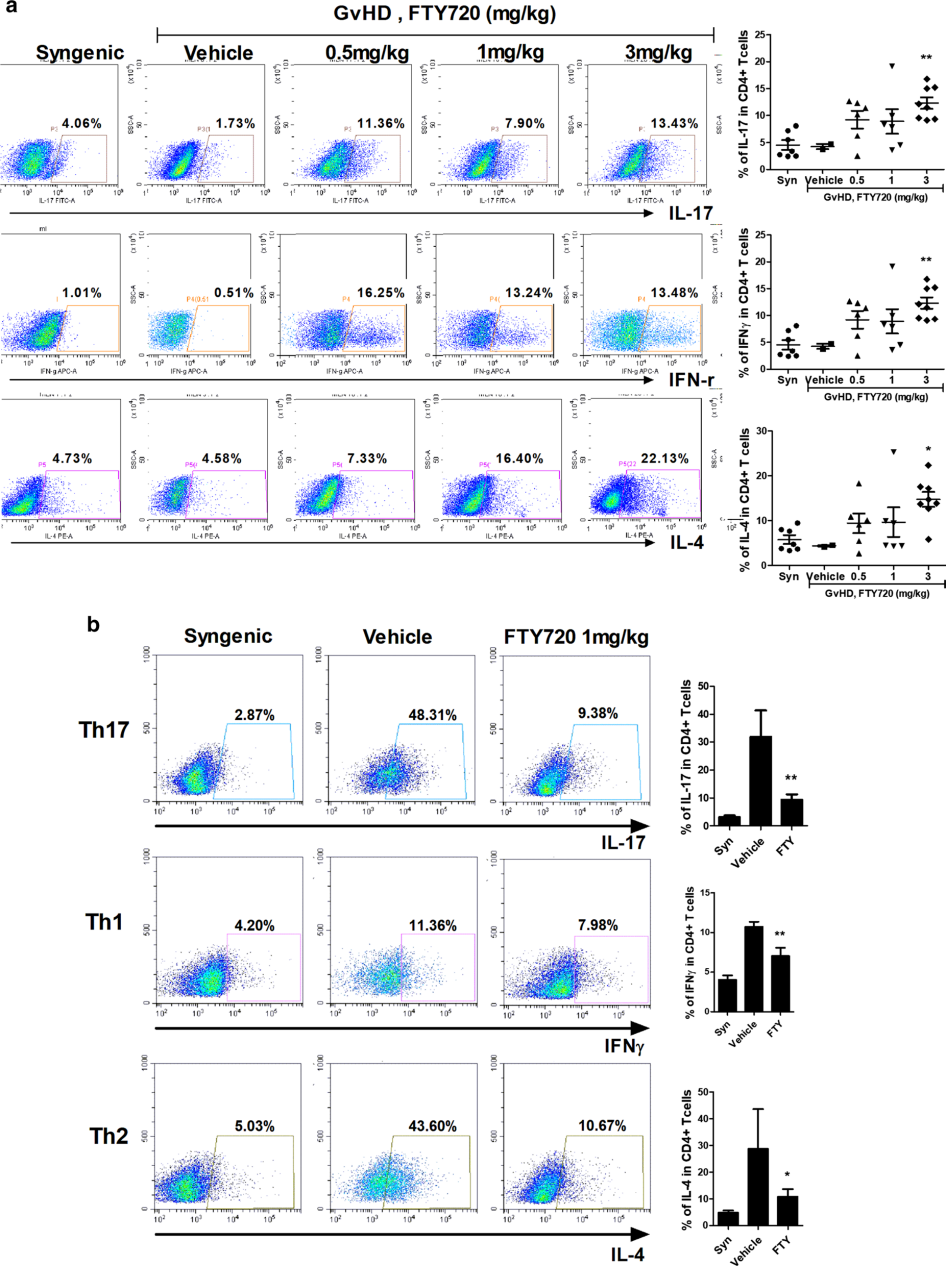
FTY720 repressed the rate of pathogenic T cells in splenocytes while FTY720 inhibited the egression of pathogenic T cells from mecenteric lymph nodes of GvHD mice. **a** The expression levels of pathogenic T cells such as IL-17, IFN-γ, and IL-4 in CD4^+^ T cells from mesenteric lymph nodes and **b** spleen in each group of mice were determined by flow cytometry. Each organs were collected on day 28 after transplantation

To ascertain the location of pathogenic T cells following FTY720 administration to GvHD mice, we conducted FACS staining. Mouse lymphocytes isolated from mesenteric lymph nodes and spleens were immunostained with fluorescently conjugated antibodies against CD4, IL-17, IFNγ, and IL-4. FTY720 upregulated IL-17, IFNγ, and IL-4 in CD4^+^ T cells from mesenteric lymph nodes in dose-dependent manner (Fig. 3a), and downregulated the same T cells from splenocytes. Especially, although the 1 mg/kg FTY720 treated-group was worse in the clinical score including weight loss than the other groups at the end of the experiment, FTY720 definitely suppressed the percentage of pathogenic T lymphocytes in the spleen from GvHD-induced mice (Fig. 3b). These results show that FTY720 blocked the escape of pathogenic T lymphocytes from mesenteric lymph nodes to the spleen.

### FTY720 restored histopathological lesions in the skin, liver, and large and small intestines

**Fig. 4:**
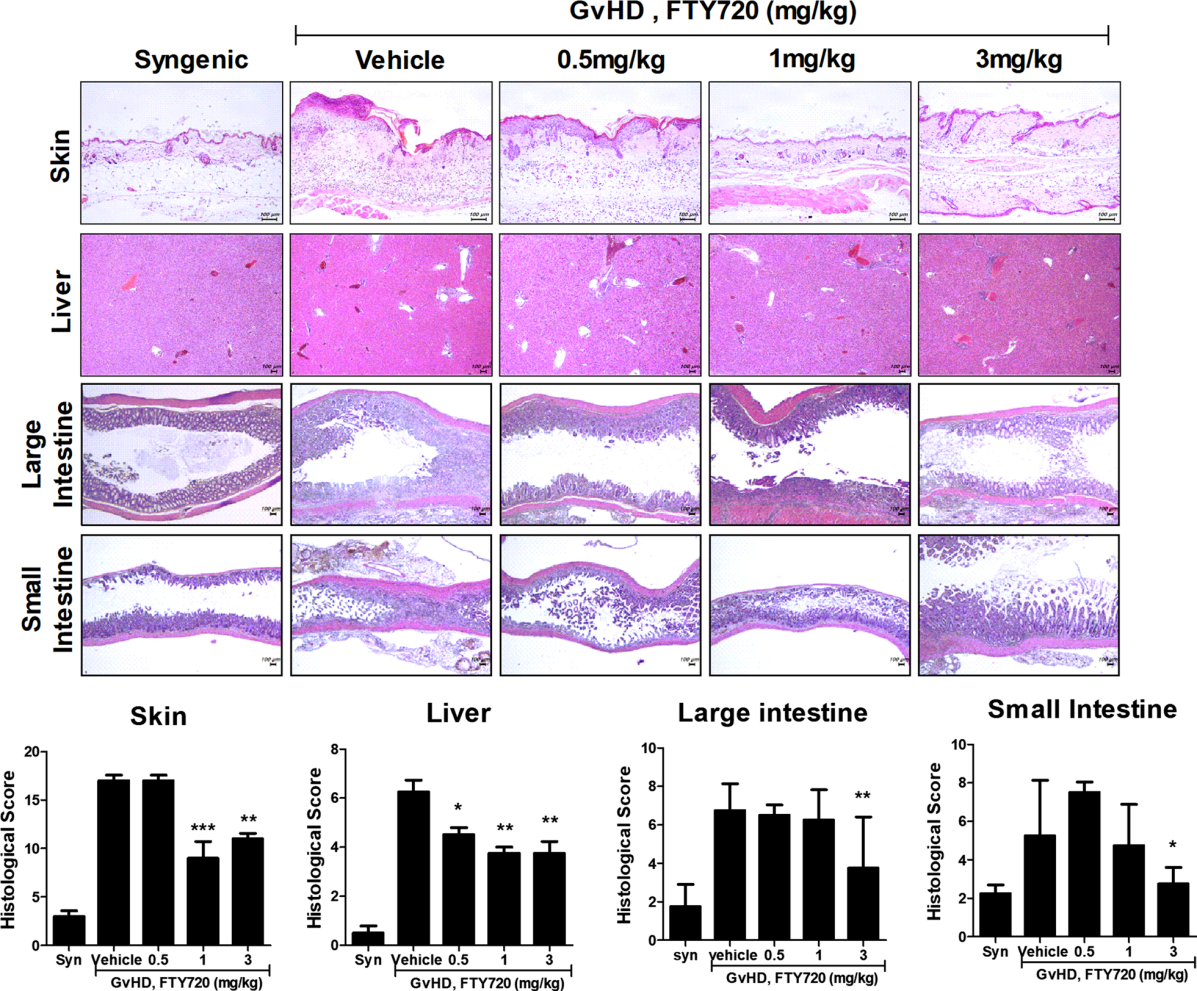
FTY720 attenuated the severity of pathogenic morphology in target organs of GvHD. Histopathology of the skin, liver and large and small intestine after BMT; the micrographs are from 1 of 2 independent experiments. The sections were stained with H&E (original magnification ×200). Epithelial loss, crypt damage, goblet cell depletion, and inflammatory cell infiltration were histologically scored. Bars represent the average histopathology scores of the skin, liver and large and small intestine, of each groups. Tissues were collected on day 28 after transplantation

FTY720 blocked the migration of T cells from mesenteric lymph nodes to the spleen (Fig. 3). Therefore, we stained skin, liver, and large and small intestinal tissue sections with hematoxylin and eosin. Although histological score of 0.5 mg/kg in FTY720-treated group was lower than vehicle group in only liver tissue, 1 mg/kg group was evaluated about half lower score than vehicle group in skin and liver tissue. Especially, 3 mg/kg group showed similar score to 1 mg/kg group in skin and liver tissue and lower score than vehicle group in large and small intestine tissue (Fig. 4). These results show that FTY720 recovered the tissue damage caused by GvHD.

### FTY720 downregulated the proliferation of fibrosis factors

**Fig. 5:**
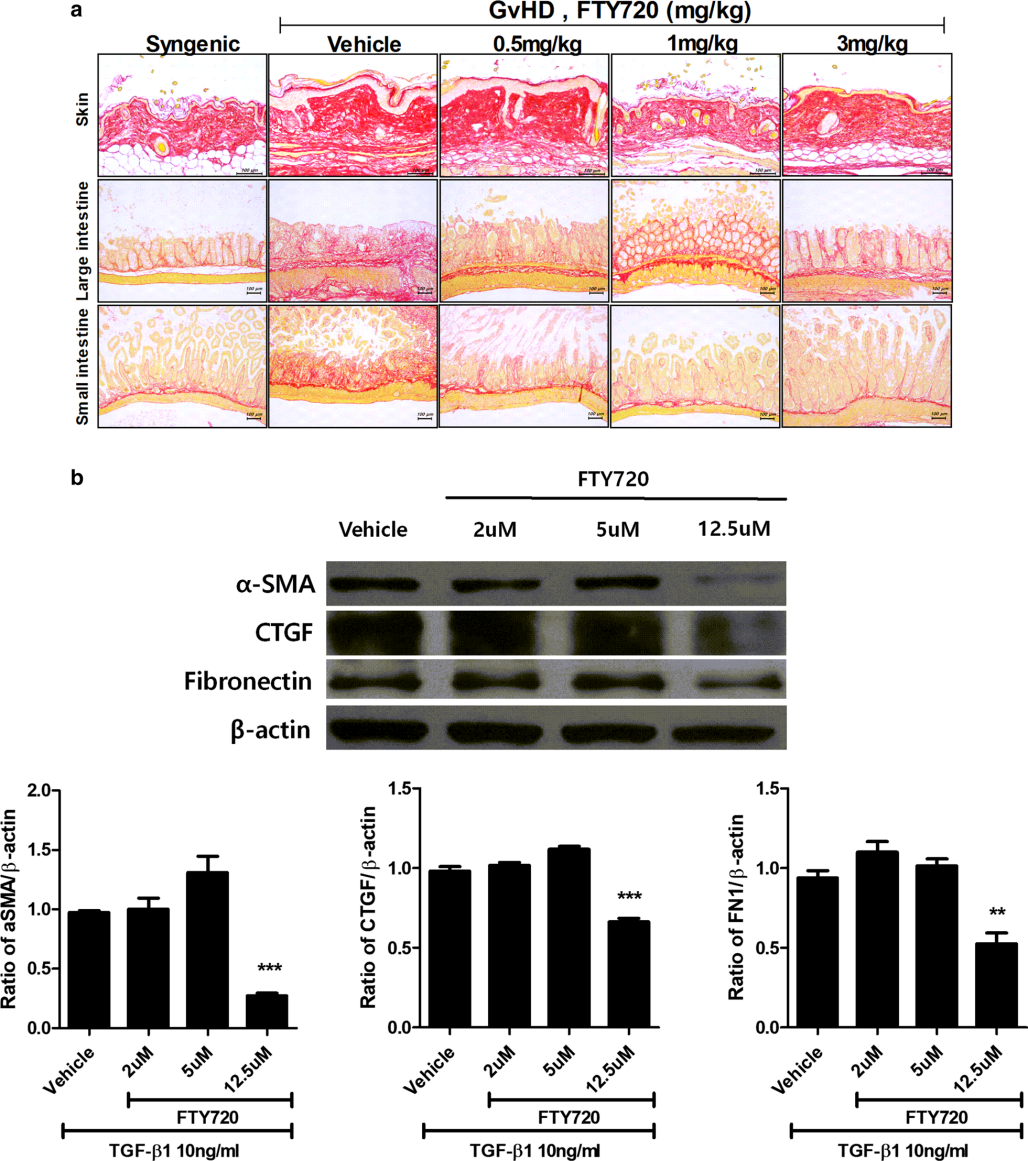
FTY720 suppressed accumulation of collagen in tissue of GvHD mice and reduced fibrosis marker protein in human skin fibroblast. **a** Immunofluorescence staining of skin, large and small intestine after BMT; the micrographs are from 1 of 2 independent experiments. The sections in paraffin molds were stained with a Picro Sirius Red Stain(original magnification ×200). Tissues were collected on day 28 after transplantation. **b** Proteins in lysates from human skin fibroblast treated with 10 ng/ml TGF-*β*1 were separated and subjected to Western blotting using the αSMA, CTGF, and Fibronectin antibodies

To investigate inhibition of fibrogenesis by FTY720, we stained skin and large and small intestinal tissues from GvHD-induced mice with sirius red, which indicates the degree of collagen accumulation in tissues. FTY720 suppressed collagen accumulation in the large and small intestinal tissues regardless of dosage, and decreased skin epidermis thickness although collagen was not reduced in the skin tissues (Fig. 5a). We therefore conducted an experiment using fibrosis in human skin fibroblasts to ascertain whether FTY720 directly regulates important fibrosis factors such as α-SMA, CTGF, and FN1. FTY720 reduced each target protein in a dose-dependent manner, to a maximum of 12.5 μM (Fig. 5b).

### FTY720 suppressed fibrogenesis in a GvHD mouse model

**Fig. 6:**
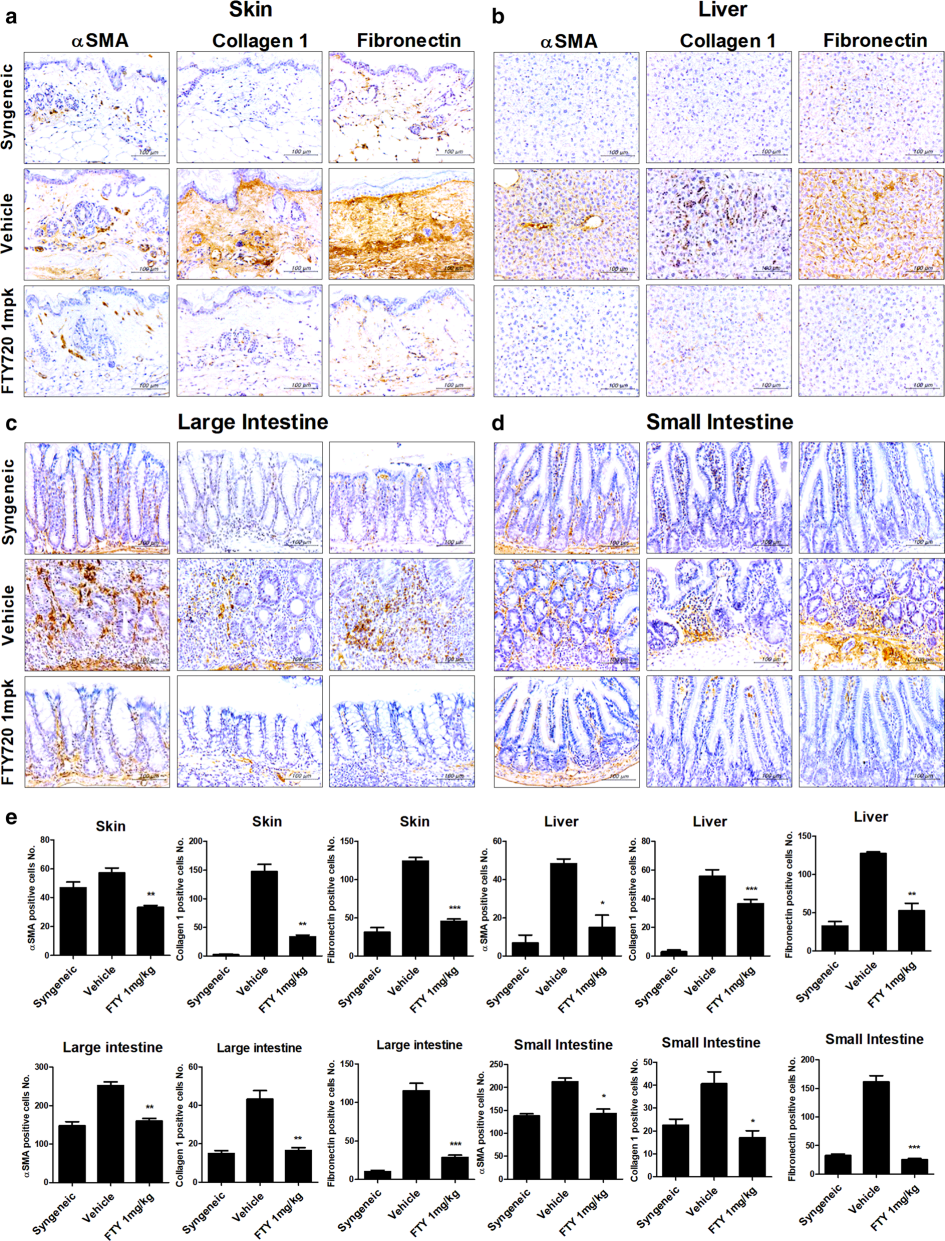
FTY720 downregulated the marker proteins of fibrosis in tissues of a murine model of GvHD. **a**–**d** Representative examples of immunohistochemical staining for αSMA, Collagen1, and Fibronectin in skin, liver, large and small intestine tissue from mice with GHVD. Positive immunoreactivity appears as a brown color, and tissues are counterstained with blue (original magnification, ×400). **e** Bars represent the average number of positive cells in the skin, liver and large and small intestine, of each groups. Tissues were collected on day 28 after transplantation

To confirm our finding that FTY720 inhibits fibrosis in a GvHD-induced mouse model, we implemented immunohistochemical staining of skin and liver tissues. Compared with the vehicle group, FTY720 downregulated fibrosis markers (α-SMA, Collagen 1, and FN1) in skin, liver, and intestinal tissues from 1 mg/kg FTY720-treated mice in FTY720-treated mice (Fig. 6a, b). These results indicate that FTY720 regulates T cell migration while simultaneously suppressing fibrogenesis.

## Discussion

Fingolimod (FTY720) is an FDA-sanctioned immunosuppressive drug applied for the therapy of a recurring and mitigating form of multiple sclerosis [9, 18, 19]. FTY720 has various biological functions including inhibition of T cell activation in a sphingosine 1 phosphate receptor-independent pathway [19, 20]. FTY720 has also been reported to inhibit T lymphocyte migration to inflammatory sites. FTY720 does not influence all lymphocytes equally [21–23]; T cells respond much more dramatically to FTY720 treatment than do B lymphocytes. Remarkably, natural killer cells are not influenced by FTY720, although they express S1P_1_, as demonstrated by reverse-transcription polymerase chain reaction (RT-PCR) [24]. Interestingly, lymphocyte response to chemokines may be affected by FTY720, since S1P commonly prevents lymphocyte over-response to chemokines [22, 25].

We then analyzed effector T cell subtypes in mesenteric lymph nodes of FTY720-treated mice. Our findings suggest that FTY720 initially prevented T lymphocyte egress from mesenteric lymph nodes. Th1, Th2, and Th17 cells were inhibited in a dose-dependent manner. Th17 cells and IL-17 have been shown to promote skin and lung inflammation and fibrosis in a bleomycin induced murine model of systemic sclerosis [26, 27]. FTY720 may affect Th17 cell populations and their trafficking through major lymphoid organs such as mesenteric lymph nodes. Th17 cells prevent eventual migration to target organs; they are thought to inhibit fibrosis in the skin, intestines, and liver. We demonstrated that FTY720 treatment significantly decreased disease severity and fibrosis in the skin and large and small intestines of a GvHD animal model.

Previous studies of FTY720 as a therapeutic drug for GvHD have focused mainly on disease models in which pathogenesis are disrupted specifically by T cells. In the current study, we examined the effect of FTY720 on a GvHD model that required effector T cells for trafficking to the target organ, and we explored the influence of this agent on fibrosis inhibition in each organ. FTY720 therapy effect a dose-dependent decrease of five clinical parameters in the GvHD animal model: weight loss, posture, activity, fur texture, and skin integrity. Our data show that T lymphocytes increased in mesenteric lymph nodes and decreased in splenocytes among FTY720-treated mice. These results are consistent with those of previous studies [9, 10, 21]. In the current study, observed symptoms of skin, liver, and intestine disease decreased in FTY720-treated mice, which showed significantly lower histopathologic scores, dermal thickness, and fibrosis area compared with the vehicle-treated group. Our immunological analysis results suggest that FTY720 treatment markedly decreased α-SMA, collagen type I, and FN1 in the skin and liver. FTY720 also decreased α-SMA, CTGF, and FN1 protein levels in keloid skin fibroblasts. The concentration of FTY720 for keloid skin fibroblasts was set up by previous study [16]. Thus, we demonstrated that oral FTY720 administration effectively reduces the severity and fibrosis of GvHD in an animal model and keloid skin fibroblasts. FTY720 suppressed the immune response by prompting Th17 cell inhibition, and reducing tissue damage and effector T cell infiltration into each organ. Recent studies have revealed many of the therapeutic effects created through another mechanisms, not the one that binds to the S1P_1_ [28–30]. These results suggest that FTY720 is a potential candidate for treatment of patients with fibrosis GvHD and we also expanded the effect of FTY720 through proving the receptor-independent effects.

In Treg cells, negative signals are carried by S1P_1_, mainly mediated by the Akt-mammalian target of the rapamycin pathway, for the development, maintenance, and function of these cells [31]. FTY720 therapy has been shown to induce an increase in Treg cells in vivo and in vitro, and to increase their suppressive function [32]. FTY720 phosphate can bind S1P_1_, resulting in the restraint of S1P_1_ signaling. Although this process reasonably explains the trafficking of effector T cells from the spleen and lymph nodes, we did not observe Treg expansion.

In the past phase III clinical trial of renal transplant, initial post hoc analysis of a population at low immunological risk revealed side-effects of FTY720 including renal dysfunction. Conversely, it was proved that FTY720 has a less toxic profile from clinical trials of MS. In particular, safety data did not reveal any nephrotoxicity. However, because the population of multiple sclerosis (MS) differed considerably from the population of renal transplant and FTY720 was used at lower doses than the renal transplant setting, clinical trials of FTY720 for transplant need to be conducted again. [33].

## Conclusion

In this study, we observed that FTY720 treatment reduced GvHD clinical scores. T lymphocytes increased in mesenteric lymph nodes and decreased in splenocytes in a FTY720-treated GvHD mouse model. Tissue analysis showed that FTY720 treatment reduced skin, intestinal inflammation, and fibrotic markers. Notably, FTY720 decreased α-SMA, CTGF, and FN1 protein levels in keloid skin fibroblasts. Thus, FTY720 suppressed the migration of pathogenic T cells to target organs, reducing inflammation. FTY720 also inhibited the expression of fibrogenesis markers in vitro and in vivo. Together, these results suggest that FTY720 prevents GvHD progression via TH17 immunosuppression and simultaneously acts as an anti-fibrotic agent. Our findings suggest that FTY720 is a potential candidate for treatment of patients with fibrosis GvHD.

## Data Availability

All data are available in the manuscript or upon request to the authors.

## Abbreviations

GvHD: Graft-versus-host disease
allo-HSCT: Allogeneic hematopoietic stem cell transplantation
APCs: Antigen-presenting cells
S1P: Sphingosine-1-phosphate
HSFs: Hypertrophic scarring fibroblasts
α-SMA: α-smooth muscle actin
BMT: Bone marrow transplantation
PBMC: Peripheral blood mononuclear cell
IL: Interleukin
IFN: Interferon
FN: Fibronectin
CTGF: Connective tissue growth factor
Th17: T helper17
Treg: Regulatory T
